# Sex similarities and differences in cognition: A longitudinal study of healthy control participants from the Parkinson Progression Markers Initiative

**DOI:** 10.1371/journal.pone.0334358

**Published:** 2025-11-24

**Authors:** Lisa Ohlhauser, Heather Kwan, Hayley Casey, Stuart MacDonald, Jodie R. Gawryluk

**Affiliations:** 1 Department of Psychology, University of Victoria, Victoria, British Columbia, Canada; 2 Institute on Aging and Lifelong Health, University of Victoria, Victoria, British Columbia, Canada; 3 Division of Medical Sciences, University of Victoria, Victoria, British Columbia, Canada; University of Thessaly Faculty of Medicine: Panepistemio Thessalias Tmema Iatrikes, GREECE

## Abstract

**Background:**

Despite sex-based differences in age-related diseases and life expectancy, limited research has explicitly examined sex differences in aging. Longitudinal study designs are particularly underutilized. The current study retrieved longitudinal data from the healthy control group of the Parkinson’s Progression Markers Initiative to examine baseline differences and cognitive changes in males and females over time.

**Methods:**

Male (n = 125, mean age = 61.61) and female (n = 68, mean age = 59.44) participants completed neuropsychological measures annually for up to five years. Measures included the Montreal Cognitive Assessment (MoCA), Letter Number Sequencing (LNS), Semantic Fluency (SFT), Symbol Digit Modalities Test (SDMT), Benton Judgment of Line Orientation Test (BJLOT), Hopkins Verbal Learning Test-Revised Immediate and Delayed Recall (HVLT-R). Within-person changes in cognition and between-group differences longitudinal change trajectories as predicted by sex were examined in a hierarchical fashion. Effects of age and education were also examined.

**Results:**

At baseline, females had higher scores on the SFT, SDMT, and the HVLT-R Immediate and Delayed Recall, while males had higher scores on the BJLOT. However, rates of change in cognition over time did not significantly differ by sex. Higher baseline age predicted lower scores for all neuropsychological outcome measures, and higher education predicted higher scores for all neuropsychological outcome measures except for the MoCA.

**Conclusions:**

Although there were sex differences in certain domains of cognitive function, rates of cognitive change over time did not significantly differ by sex. Intraindividual variability in cognitive trajectories of aging was observed. Future research should examine factors that predict individual trajectories of aging in healthy individuals.

## Introduction

The global population of adults over the age of 65 is expected to double from 703 million to 1.5 billion by 2050 [1]. The shift in population demographics emphasizes the need to understand normal age-related changes in cognition, to aid in promoting health and longevity into the later decades of life. Although there are differences between males and females in life expectancy and age-related diseases, longitudinal studies investigating sex differences among healthy individuals has been limited.

One challenge within the literature is the bidirectional entanglement of terminology and concepts whereby it is extremely challenging to differentiate inborn sex factors from social and environmental gender learning [2]. Sex/gender is a term that has been used in the literature when discussing the difficulty of separating these variable or when the terms have not been adequately defined [3,4] and will be used herein. Most of the literature to date has examined cognitive aging without investigating differences in sex/gender. However, these are important factors that may influence cognitive aging trajectories, especially given sex-based differences in longevity and predisposition to neurodegenerative conditions [5].

The “gender similarities hypothesis” posits that males and females are largely similar on most, but not all variables [6]. Although males and females are more similar cognitively than they are different, questions remain about sex/gender differences in the normal aging trajectory. Significant sex/gender differences have been found in specific cognitive abilities, including visuospatial working memory [7], mental rotation [8], episodic memory [9], and verbal abilities [10,11]; however, the effect sizes of these differences are usually quite small [6]. Overall, most longitudinal studies have found minimal sex/gender differences in the rate of cognitive changes over time in older healthy adults. A meta-analysis of 13 longitudinal studies concluded that sex/gender was not a determining factor in cognitive decline amongst individuals aged 60–80 years [12]. Although the meta-analysis did not find sex/gender differences in overall cognitive level, females outperformed males on tests of episodic memory [13,14] and males outperformed females on tests of visuospatial ability [13–15]. Sex/gender differences for tests of semantic memory and attention were more inconsistent [12]. The overall evidence suggested that there are few if any sex/gender differences in cognitive aging in healthy older adults before age 80 and that other factors, such age, education, and social behaviour were found to be more predictive of cognitive changes than sex/gender [12]. However, other studies, such as the Baltimore Longitudinal Study on Aging, have found steeper rates of decline for males on measures of mental status, perceptuomotor speed and integration, and visuospatial ability, suggesting that females may have a higher resilience to age-related cognitive decline compared to males [16]. Specifically, females showed higher baseline scores on tests of verbal learning and memory, fluent language production, mental status, and psychomotor speed, while males showed an advantage on tests of visuospatial ability. Both males and females showed a decline on all cognitive measures over the study period of 3–9 years [16].

Despite extant studies, more research is needed to examine trajectories of aging as a function of sex and longitudinal study designs are necessary for measuring intraindividual change over time in healthy adults. The objective of the present study was to use data from the healthy cohort of the Parkinsons Progression Markers Initiative (PPMI) to answer three primary research questions, as follows:

1) Are there changes in cognitive performance that occur among healthy adults over a five-year time frame?
2) Are there sex differences in cognitive performance in healthy adults?
3) Are there sex differences in rates of change in cognition over five years of healthy aging?

Based on previous literature, it was hypothesized that most individuals would be stable or show small decreases in scores on the neuropsychological measures of processing speed, verbal fluency, and some aspects of executive functioning and memory [12], while vocabulary would remain more stable [17]. Consistent with previous research [13,14], females were expected to score higher on verbal measures (i.e., Semantic Fluency Test, Hopkins Verbal Learning Test – Revised) and males were predicted so score higher on visuospatial measures (i.e., Judgment of Line Orientation). Finally, given the subtle sex differences in verbal fluency that have been found in previous research, it was hypothesized that men may experience greater decline on the verbal fluency relative to women. Other domains were predicted to change similarly between the sexes.

## Methods

### Participants

Data were retrieved from the Parkinson’s Progression Markers Initiative (PPMI) database (www.ppmi-info.org/data) corresponding to 01/04/2020. The mean age for male participants at baseline (n = 125) was 61.6 years (SD = 10.9, Range = 30.61 to 82.25) and was 59.4 years (SD = 11.6, Range = 31.86 to 81.79) for females (n = 68). For up-to-date information on the PPMI, which began recuiting participants in 2010, visit www.ppmi-info.org. PPMI – a public-private partnership – is funded by the Michael J. Fox Foundation for Parkinson’s Research and funding partners, which can be found at www.ppmi-info.org/fundingpartners. Participants from the PPMI were eligible for inclusion in the present study if: a) they were classified as a healthy control group participant by PPMI (this included, (1) Ability to provide written informed consent in accordance with Good Clinical Practice (GCP), International Conference on Harmonization (ICH), and local regulations. (2) Willing and able to comply with scheduled visits, required study procedures and laboratory tests. (3) Women may not be pregnant, lactating, or planning pregnancy during the course of the study. (4) Male or female age 30 years or older at screening.) and b) neuropsychological data was available for at least one time point. Up to six annual time points were available (i.e., baseline or time 0, time 1, 2, 3, 4, 5). Participants were excluded from the present analysis if PPMI’s criteria for a healthy control participant was not met at any time point, overall this included, (1) Received any of the following drugs that might interfere with dopamine transporter SPECT imaging: Neuroleptics, metoclopramide, alpha methyldopa, methylphenidate, reserpine, or amphetamine derivative, within 6 months of Screening. (2) Current treatment with anticoagulants (e.g., coumadin, heparin) that might preclude safe completion of the lumbar puncture. (3) Condition that precludes the safe performance of routine lumbar puncture, such as prohibitive lumbar spinal disease, bleeding diathesis, or clinically significant coagulopathy or thrombocytopenia. (4) Any other medical or psychiatric condition or lab abnormality, which in the opinion of the investigator might preclude participation. (5) Use of investigational drugs or devices within 60 days prior to baseline (dietary supplements taken outside of a clinical trial are not exclusionary, e.g., coenzyme Q10). (6) Previously obtained MRI scan with evidence of clinically significant neurological disorder (in the opinion of the Investigator). (7) Current or active clinically significant neurological disorder (in the opinion of the Investigator). (8) First degree relative with idiopathic Parkingon’s disease (parent, sibling, child). (9) MoCA score less than or equal to 26. Additional exclusion criteria was applied at individual timepoints including, as two or more neuropsychological test scores < 1.5 standard deviations below the mean or a PPMI investigator rating of mild cognitive impairment or dementia.

It is important to note that the PPMI Operations Manual indicated that a sex/gender variable was based on “gender at birth”. Based on the consensus definitions in the literature [18,19] it is apparent that PPMI’s use of “gender” corresponds with the consensus definitions of “sex” and this variable was treated as such. The PPMI did not collect data corresponding to the consensus definitions of “gender”, so it was not possible to examine the effects of gender within this database. Ethical approval for the original data collection was obtained by the PPMI investigators; consent was obtained for all participants and research was completed in accordance with the Helsinki Declaration. Ethical approval for secondary data analysis for the present study was obtained from the University of Victoria Human Ethics Board.MeasuresNeuropsychological data were collected by PPMI researchers at baseline (time 0) and then approximately annually for five years, for up to a total of six annual time points (i.e., baseline or time 0, 1, 2, 3, 4, 5). The present study utilized scores on six neuropsychological measures, including the Montreal Cognitive Assessment (MoCA), the Letter Number Sequencing test (LNS), the Semantic Fluency Test (SFT), the Symbol Digits Modalities Test (SDMT), the Benton Judgement of Line Orientation Test (BJLOT), and the Hopkins Verbal Learning Test – Revised (HVLT-R). A brief description of each measure and the specific scores used as outcome measures is summarized in Table 1.

**Table 1:** Description of neuropsychological measures and cognitive domains assessed.

Neuropsychological Measure	Cognitive Domain(s) Assessed	Description of Measure	Score Calculation	Repeated Measurements
Montreal Cognitive Assessment (MoCA)	Overall cognitive ability	A screener for mild cognitive impairment and dementia. Brief assessment of visuospatial/executive, naming, memory, attention, language, abstraction, and orientation.	Sum of correct items (maximum score = 30)	Same form at each time point.
Letter Number Sequencing (LNS)	Working memory	Sequence a string of letters and numbers of increasing length alphabetically and numerically.	Sum of correctly sequenced trials (maximum score = 21)	Same form at each time point.
Semantic Fluency Test (SFT)	Verbal fluency, executive functioning, and memory retrieval	Name as many items in a category in one minute.	Sum of three categories (no maximum score)	Same categories at each time point.
Symbol Digit Modalities (SDMT)	Executive functioning, working memory, and processing speed	Write numbers to match symbols using a key.	Number of correctly coded symbols. (maximum score = 110)	Alternated between Form 1 and Form 2 for each time point.
Benton Judgement of Line Orientation (BJLOT)	Visuospatial ability	Judge the angle of lines by matching them to a key.	Number of correctly identified line orientations (maximum score = 15)	Alternated between odd and even items at each time point.
Hopkins Verbal Learning Test – Revised (HVLT-R)	Verbal learning and memory	Learn a list of unrelated words over three learning trials, then recall it freely after a delay.	Immediate Recall is the sum of three learning trials (maximum score = 36) Delayed Recall is the sum of correctly recalled words after a 20-minute delay (maximum score = 12)	Unique form for each time point (i.e., 6 unique word lists)

### Study design

**Fig 1:**
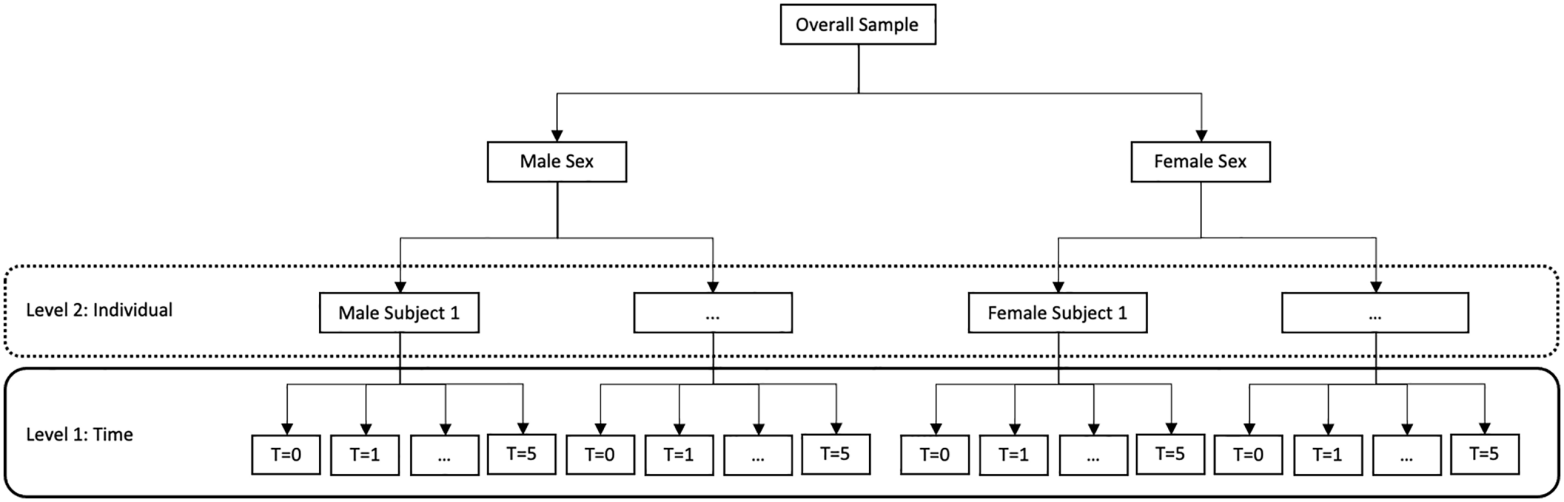
Schematic of the 2-level linear mixed model. Each neuropsychological score was treated as a dependent variable and modelled separately. Level-1 represented within-person repeated measurements in cognition for up to six time points and level-2 represented between-person differences based on several time-invariant predictors. This figure displays the primary focus of the present study on evaluating between-person differences in cognitive change over time as predicted by the categorical predictor of sex (i.e., male or female). Other time-invariant level-2 predictors investigated were years of education and age at baseline.

A series of 2-level linear mixed models were created using each of the seven neuropsychological scores as a single dependent or outcome variable in seven separate models (i.e., MoCA, SDMT, LNS, SFT, BJLOT, HVLT-R immediate and delayed). These models were used to estimate within-person change in cognition over time (i.e., level-1, repeated measures or time level), between-person differences in cognition by sex, and between-person differences change trajectories as predicted by sex (i.e., level-2, individual level). The structure of the 2-level design is displayed in Fig 1, where repeated measures are nested within individuals. Additional level-2 time-invariant predictors were also investigated, including baseline age and education. Model specification and equations are shown in Table 2.

**Table 2:** Multi-level model specification equations to predict neuropsychological outcome measures.

	Model		
Components	Unconditional: Random intercept only model	Unconditional Growth	Conditional Growth
Level-1 Equation	${NP}_{ti}=\beta_{\text{0}i}+e_{ti}$	${NP}_{ti}=\beta_{\text{0}i}+\beta_{\text{1}i}{TIME}_{ti}+e_{ti}$	${NP}_{ti}=\beta_{\text{0}i}+\beta_{\text{1}i}{TIME}_{ti}+e_{ti}$
Level-2 Equation (s)	$\beta_{\text{0}i}=\gamma_{\text{0}\text{0}}+U_{\text{0}i}$	$\beta_{\text{0}i}=\gamma_{\text{0}\text{0}}+U_{\text{0}i}$ $\beta_{\text{1}i}=\gamma_{\text{1}\text{0}}+U_{\text{1}i}$	$\beta_{\text{0}i}=\gamma_{\text{0}\text{0}}+{\gamma_{\text{0}\text{1}}X_{\text{1}}+U}_{\text{0}i}$ $\beta_{\text{1}i}=\gamma_{\text{1}\text{0}}{\;+\;\gamma }_{\text{1}\text{1}}X_{\text{1}}+U_{\text{1}i}$
Composite Equation	${NP}_{ti}=\gamma_{\text{0}\text{0}}+U_{\text{0}i}+e_{ti}$	${NP}_{ti}=\;\gamma_{\text{0}\text{0}}+U_{\text{0}i}$ $+\gamma_{\text{1}\text{0}}{TIME}_{ti}+U_{\text{1}i}{TIME}_{ti}+e_{ti}$	${NP}_{ti}={\;\gamma }_{\text{0}\text{0}}+{{\;\gamma }_{\text{0}\text{1}}X}_{\text{1}}+{\;\gamma }_{\text{1}\text{0}}{{*TIME}_{ti}\;+\;\gamma }_{\text{1}\text{1}}X_{\text{1}}*{TIME}_{ti}{\;+\;U}_{\text{0}i}+U_{\text{1}i}*{TIME}_{ti}+e_{ti}$
R-syntax: lmer(y ~ …	1 + (1 | ID)	time + (time | ID)	time * *X*_1_ + (time | ID)
Predictors: Level-1		${TIME}_{ti}$ = time in years between measurement occasions (continuous; centered on the baseline assessment), where *t* = exact time in years since the baseline assessment for individual participant, *i*. Data were unbalanced, meaning participants had 1–6 measurement occasions.	
Predictors: Level-2			*X*_1_ = sex (categorical; 0 = female, 1 = male) *X*_2_ = baseline age (continuous; centered on mean baseline age) *X*_3_ = years of education (continuous; centered on mean education)
Interactions			Time*Sex Time*Age

*Note.* Dependent variables were scores on neuropsychological measures (*NP*), which were modeled separately: Montreal Cognitive Assessment – Total Score/30; Letter Number Sequencing – Total Score/21; Semantic Fluency – Total Score (Sum of Animals, Vegetables, and Fruits); Symbol Digit Modalities Test – Total Score; Hopkins Verbal Learning Test – Revised Immediate Recall (Sum of Learning Trials 1–3)/36 & Delayed Recall/12; Benton Judgement of Line Orientation Test/15.

### Procedure and data analysis

Data for the healthy control cohort was retrieved from the PPMI website, specifically from the file labeled “PPMI_Original_Cohort_BL_to_Year_5_Dataset_Apr2020.csv”. Data analyses were conducted using the statistical software R and R Studio. The R package for Linear Mixed Effects Models using ‘Eigen’ and S4 (i.e., “lme4”) was utilized for the multi-level linear modeling [20]. Models were then compared using the anova command from the “lmerTest” package for R, which produced type I, II, and III anova tables for fixed-effect terms with Satterthwaite and Kenward-Roger methods for denominator degrees of freedom for F-tests. Akaine Information Criterion (AIC) and the Bayesian Information Criterion (BIC) were examined to determine the final model fit for the data.

## Results

**Fig 2:**
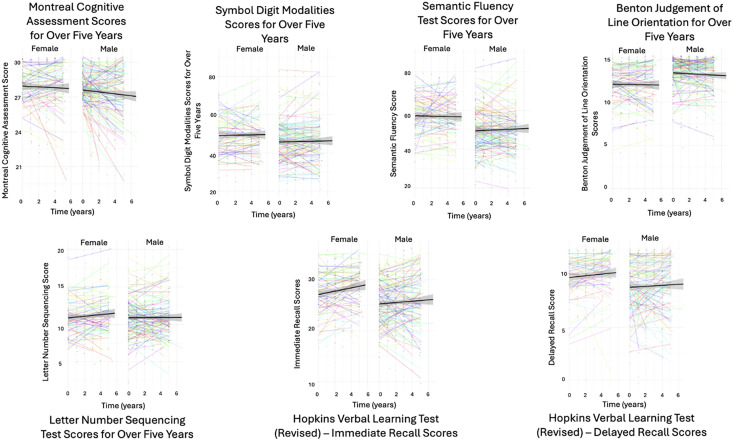
Cognitive Assessment Scores over time. Coloured lines show individual growth trajectories over time. Black lines show the mean change in scores over time by sex with the shaded portions showing the standard error in the estimate. Individual points are slightly shifted and may appear higher than the maximum score for the purpose of visualizing multiple data points.

Descriptive statistics including the number of participants, sex, and race/ethnicity at each timepoint, along with the means and standard deviations for the participants’ ages, years of education, and neuropsychological test scores are shown in Table 3. Notably, males and females did not significantly differ in terms of age at baseline (males 61.6 years, females 59.4 years, p = 0.19), although males were significantly more educated than females (males 16.4 years, females 15.5 years, p = 0.03). Spaghetti plots were graphed to show the neuropsychological scores for each participant across all timepoints using the R package “ggplot2” (Fig 2). Multilevel data analysis results are presented using the recommended Logical Explanations and Visualizations of Estimates in Linear mixed models (LEVEL) checklist [21]. A summary of the final effect estimates for all seven neuropsychological outcome measures (which were modeled separately) is displayed in Table 4. Although the fit of the final models varied slightly depending on the neuropsychological outcome measure, the final models with all the predictors (i.e., sex, age, and education) and interactions of interest (i.e., time by sex, time by age) are shown.

**Table 3:** Participant demographics and descriptive statistics.

		Time Point					
Mean (SD) or n		Baseline or 0	1	2	3	4	5
Years since baseline (SD)		NA	1.04 (0.08)	2.03 (0.10)	3.02 (0.09)	4.03 (0.09)	5.05 (0.18)
Total N		193	182	166	160	155	145
Race/Ethnicity	White	178	169	154	148	144	138
	Black	10	9	8	8	8	6
	Asian	1	1	1	1	0	0
	Other	4	3	3	3	3	1
Sex	Female	68	66	63	61	59	57
	Male	125	116	103	99	96	88
Age	Female	59.4 (11.6)	60.7 (11.5)	61.4 (12.0)	63.0 (11.8)	63.8 (12.0)	63.6 (11.3)
	Male	61.6 (10.9)	62.9 (10.9)	63.2 (11.2)	64.5 (10.7)	65.5 (10.6)	65.7 (10.5)
	Range	30.6-83.7	31.9-85.3	32.9-83.9	33.9-85.0	34.9-85.8	34.1-87.7
Education	Female	15.5 (2.7)	15.6 (2.7)	15.5 (2.7)	15.6 (2.8)	15.6 (2.8)	15.8 (2.8)
	Male	16.4 (2.9)	16.3 (2.9)	16.4 (2.9)	16.3 (3.0)	16.4 (3.1)	16.4 (3.0)
MoCA	Female	28.3 (1.1)	27.5 (2.2)	27.8 (3.9)	27.7 (2.1)	27.7 (2.4)	28.0 (2.1)
	Male	28.2 (1.1)	27.1 (2.2)	26.8 (2.5)	27.3 (2.1)	27.5 (2.3)	27.4 (2.2)
LNS	Female	10.9 (2.4)	10.9 (2.9)	11.1 (2.6)	11.1 (2.9)	11.3 (2.7)	11.5 (2.9)
	Male	10.8 (2.7)	11.0 (2.7)	10.9 (2.5)	11.0 (2.7)	10.9 (2.8)	10.9 (2.8)
SFT	Female	57.2 (10.7)	57.1 (9.8)	57.4 (11.5)	57.1 (11.0)	55.7 (10.5)	57.8 (11.4)
	Male	49.1 (10.5)	50.0 (11.2)	50.3 (11.3)	50.3 (11.5)	49.7 (11.8)	50.7 (11.7)
SDMT	Female	48.9 (10.2)	49.9 (10.1)	48.6 (10.2)	49.9 (9.8)	48.6 (10.0)	50.2 (10.3)
	Male	45.6 (10.7)	46.2 (11.4)	45.5 (11.2)	46.8 (11.8)	45.3 (11.7)	46.6 (12.3)
BJLOT	Female	12.4 (2.2)	11.6 (2.6)	12.4 (2.4)	11.7 (2.5)	12.4 (2.8)	11.9 (2.6)
	Male	13.5 (1.8)	13.2 (2.2)	13.5 (1.8)	13.1 (2.1)	13.2 (2.2)	13.3 (1.9)
HVLT-R							
*Immediate Recall*	Female	27.2 (4.4)	27.6 (4.5)	27.8 (4.8)	28.0 (4.8)	27.6 (4.5)	29.4 (3.8)
	Male	25.3 (4.4)	25.7 (4.7)	24.9 (4.8)	25.8 (5.3)	25.5 (5.4)	26.3 (5.8)
*Delayed Recall*	Female	9.9 (1.9)	9.7 (2.2)	10.1 (2.2)	9.6 (2.2)	9.9 (2.2)	10.4 (2.2)
	Male	8.9 (2.5)	8.8 (2.6)	8.7 (2.6)	9.0 (2.6)	8.7 (2.5)	9.4 (2.8)

*Note.* MoCA = Montreal Cognitive Assessment, LNS = Letter Number Sequencing, SFT = Semantic Fluency, SDMT = Symbol Digit Modalities Test, BJLOT = Benton Judgement of Line Orientation, HVLT = Hopkins Verbal Learning Test Revised, IR = Immediate Recall (Sum of 3 learning trials), DR = delayed recall.

**Table 4:** Summary of the multi-level models to predict cognition on seven difference neuropsychological outcome measures over five years.

	Estimate (Confidence Interval)						
Outcome Measure	(Intercept)	Time	Sex	Age	Education	Time x Sex	Time x Age
MoCA	27.92*** **(27.59–28.24)**	−0.04 (−0.15–0.06)	−0.24 (−0.65–0.16)	−0.03*** **(−0.05 – −0.02)**	0.01 (−0.06–0.07)	−0.08 (−0.21–0.06)	−0.01** **(−0.01–0.00)**
LNS	10.88*** **(10.36–11.39)**	0.05 (−0.05–0.16)	0.08 (−0.56–0.73)	−0.08*** **(−0.11 – −0.05)**	0.15** **(0.05–0.25)**	−0.11 (−0.24–0.03)	0 (−0.01–0.00)
SFT	57.04*** **(54.73–59.35)**	−0.04 (−0.43–0.36)	−7.29*** **(−10.19 – −4.39)**	−0.23*** **(−0.35 – −0.10)**	0.51* **(0.04–0.98)**	−0.02 (−0.53–0.48)	−0.01 (−0.03–0.01)
SDMT	48.94*** **(47.01–50.87)**	0.06 (−0.31–0.42)	−2.76* **(−5.18 – −0.33)**	−0.5*** **(−0.60 – −0.40)**	0.71*** **(0.33–1.09)**	−0.05 (−0.51–0.42)	−0.01 (−0.03–0.01)
BJLOT	12.2*** **(11.78–12.63)**	−0.03 (−0.12–0.06)	1.21*** **(0.68–1.74)**	−0.03* **(−0.05 – −0.00)**	0.15*** **(0.07–0.23)**	−0.03 (−0.14–0.09)	−0.01* **(−0.01 – −0.00)**
HVLT-R Immediate Recall	27.17*** **(26.24–28.09)**	0.25* **(0.05–0.45)**	−1.85** **(−3.01 – −0.70)**	−0.1*** **(−0.15 – −0.05)**	0.28** **(0.10–0.47)**	−0.19 (−0.44–0.07)	−0.01 (−0.02–0.00)
HVLT-R Delayed Recall	9.77*** **(9.29–10.24)**	0.05 (−0.05–0.15)	−0.93** **(−1.52 – −0.33)**	−0.05*** **(−0.08 – −0.03)**	0.13** **(0.04–0.22)**	−0.02 (−0.15–0.11)	0 (−0.01–0.00)

*Note.* MoCA = Montreal Cognitive Assessment, LNS = Letter Number Sequencing, SFT = Semantic Fluency, SDMT = Symbol Digit Modalities Test, BJLOT = Benton Judgement of Line Orientation, HVLT = Hopkins Verbal Learning Test Revised, IR = Immediate Recall (Sum of 3 learning trials), DR = delayed recall. Time, age, and education were centered so that time at baseline, mean baseline age of 60.62, and mean years of education of 16.07 were equal to 0. For sex, positive values indicate a male advantage and negative values indicate a female advantage. ***p < .001. **p < .01. *p < .05.

### Results for Question 1: What are the cognitive changes that occur among healthy adults over a five-year time frame (i.e., level-1, within-person change)?

All neuropsychological outcome measures, except for HVLT-R Immediate Recall, showed stability in scores within-individuals over time (i.e., no systematic change among all participants). On the HVLT-R Immediate Recall, individuals showed a slight improvement over time, by approximately 0.25 words per year.

#### Interaction of time and age.

The interaction between change in neuropsychological scores over time and baseline age was non-significant for the LNS, SFT, SDMT, and the HVLT-R Immediate and Delayed. In the case of the MoCA and the BJLOT, the estimates were extraordinarily small, but indicated greater rates of decline in MoCA (−0.01) and BJLOT (−0.01) scores over time for each year above the baseline mean age of approximately 60 years. All other measures showed no differences in rates of neuropsychological scores over time based on age at the baseline assessment.

### Results for Questions 2: Are there sex differences in cognition (i.e., level-2, differences in intercepts)?

At baseline, females had higher scores on the SFT (−7.29), SDMT (−2.76), and the HVLT-R Immediate (−1.85) and Delayed Recall (−0.93), while males had higher scores on the BJLOT (1.21). Scores on the MoCA and the LNS were balanced at all time points.

Baseline age was a significant predictor of cognition for all neuropsychological outcome measures, where higher age at baseline was reflective of lower cognitive performance.

Education was also a significant predictor in all models, except for the MoCA. Higher years of education was predictive of higher cognitive performance (estimates ranged from −0.08 to −0.23).

### Results for Question 3: Are there sex differences in rates of change in cognition over five years (i.e., level-2, between-group differences in change, differences in slopes)?

#### Interaction of time and sex.

The interaction between change in neuropsychological scores over time and sex was non-significant in all models. Males and females did not systematically differ in their rates of change in any of the neuropsychological scores over time (estimates ranged from – 0.02 to −0.19).

## Discussion

The current study contributes to a growing body of literature aimed at investigating expected cognitive changes due to normal aging and how these changes may be similar or different by biological sex. Data were sourced from the healthy control group from the PPMI. Annual cognitive changes were examined for five years (i.e., up to six time points) in 193 healthy adult males and females with an aim to answer three primary research questions: 1) what are the cognitive changes that occur in healthy older adults over five years, 2) what are the sex differences in cognition, and 3) what are the sex differences in rates of cognitive change over five years? The findings in the context of each of these questions are discussed below.

### Finding 1: Stability in neuropsychological scores over time

Regarding the first question of cognitive changes over time, no systematic within-person change was found on any of the neuropsychological outcome measures, except for the HVLT-R Immediate Recall. On the HVLT-R Immediate Recall, slight increases in scores were observed over time. These results suggest overall stability among almost all neuropsychological scores in this healthy adult sample that was followed over five years.

These findings are consistent with cross-sectional and longitudinal findings of cognition in a robust sample of over 26,000 participants from the UK Biobank, which found minimal changes in cognition prior to the age of 65 [22]. Even though many longitudinal studies of aging in older adults free of dementia have found overall decline in cognitive performance over time [16,23–26], some have found overall stability in cognitive performance. For example, in the Canadian Study of Health and Aging, almost 50% of participants over the age of 65 showed virtually no change in cognition over 10 years [27]. In the present study, the interaction effect of age and did not reveal significant differences. The present sample included the entire healthy control cohort of the PPMI, which had a wide age range at baseline from 30.63 to 83.68 years. Participants in the present sample were excluded from all time points of the healthy aging cohort by PPMI investigators if performance was more than 1.5 standard deviations below the mean on two or more neuropsychological test scores or there was a PPMI investigator rating of mild cognitive impairment or dementia. Given these exclusion criteria, scores were constrained to fall within the normal range based on pre-existing normative data.

There was a slight increase in HVLT-R Immediate Recall scores over time. Significant increases in HVLT-R scores over one year were also found in a sample of over 16,000 healthy older community dwelling adults with an approximate mean age of 75 from the ASPirin in Reducing Events in the Elderly (ASPREE) study who were tested with the same form [28]. Retest effects in individuals in young to middle adulthood have been demonstrated to be quite strong, such that retest effects may mask age effects or even exceed them to show positive changes over time [29,30]. Indeed, other longitudinal studies have observed improvements in cognitive measures over time [22], including other verbal memory measures, such as the California Verbal Learning Test, Second Edition [31,32]. Alternative forms of the HVLT-R were used at each assessment in the present study, which have demonstrated acceptable reliability to the original form [33]. However, significant practice effects in serial neuropsychological testing have been found for similar verbal memory measures, such as the Rey Auditory Verbal Learning Test, even when using alternative forms [34].

Although virtually no systematic change in neuropsychological scores was detected in the current study, examination of the spaghetti plots (Fig 2) revealed great intraindividual variability in scores over time. Some individuals appeared to have decreases in scores over time, while others appeared to show stability or increases in scores. The intraclass correlation coefficients for the neuropsychological outcome measures ranged from 0.58 to 0.75, confirming highly correlated scores within-persons over time and larger between-person variance in scores. This may have created a “fanning” effect that ameliorated overall systematic change over time. These findings underscore the importance of examining factors that might explain intraindividual variability in aging trajectories. For example, emerging work has strived to categorize healthy individuals into non-overlapping cognitive phenotypes based on baseline factors (e.g., demographics, psychological measures, plasma cytokines, and neuroimaging variables) and varying change trajectories within the normal range in certain cognitive domains to predict vulnerabilities in aging in healthy high functioning adults [35].

In the current study, several factors to explain these between-person differences in trajectories were examined. Consistent with findings in the literature (reviewed in Sanchez-Izquierdo & Fernandez-Ballesteros, 2021), higher age and lower education at baseline predicted lower performance on most neuropsychological measures, with the exception of the MoCA. There was also a statistically significant time by age interaction for the models predicting MoCA and BJLOT scores. Although these estimates were statistically significant, they were exceedingly small and unlikely to be clinically meaningful. Another primary research question examined whether there were sex differences in cognition and how rates of change may differ by sex.

### Finding 2: Sex difference in neuropsychological scores at baseline

Significant sex differences on several neuropsychological measures were detected. Specifically, females scored higher on the SFT, the HVLT-R Immediate and Delayed Recall, and the SDMT, reflecting higher performance in verbal fluency, verbal learning and memory, and a multifaceted measure of attention, working memory, and information processing speed. Males scored higher on the BJLOT, reflecting higher performance in visuospatial ability. Males and females did not significantly differ on the MoCA or the LNS, reflecting similar performances in global cognitive functioning and auditory working memory.

These findings are consistent with the literature. There is a lengthy and well-documented female advantage for performance on verbal tasks, including verbal fluency and memory [36,37]. Recent meta-analyses support a small female advantage in verbally based episodic memory tasks [9,38], a very small to negligible female advantage for verbal working memory [39], and female advantage in phonemic but not semantic fluency tasks, which appear to be category dependent [38]. Given this literature, it is not surprising that females scored higher than males on the SFT, which was the sum of each number of category words named in one minute where there is a known advantage for females in two out of three of the categories assessed (i.e., vegetables and fruit) [38]. Further, females scored higher on a verbal memory measure – the HVLT-R Immediate and Delayed Recall. Although the originally published normative data for the HVLT-R included 541 healthy adults aged 17–88 from the United States provided scores stratified only on age [33,40], recently expanded normative data for older adults aged 65 included additional variables and demonstrated that females consistently scored higher than males on all components of the HVLT-R [41]. Females also scored almost three points higher than males at baseline on the SDMT. This finding is also consistent with updated normative data, where females have demonstrated higher scores than males for both the oral [42] and written version of the test [43]. Finally, males scored higher than females on the BJLOT, which is consistent with the finding of stronger performance on visuospatial ability in males compared to females (Halpern et al., 2007 [44]; meta-analysis in Voyer et al., 2017). Corrections for age and sex are typically made for the BJLOT, where males have been found to score approximately 2 points higher than females on both the full and short forms (described in Strauss et al., 2006 [45]), which was very comparable to the present findings. Similar sex differences in cognitive abilities at baseline time points have been found in several longitudinal studies of healthy aging [13,16,22,46].

The reasons for sex differences in cognitive abilities are complex and likely influenced by multiple factors. Biologically, sex hormones are thought to interact with cognition. For example, contraceptives have been linked with higher performance on verbal fluency and mental rotations tasks [47]. Sex hormones, such as estrogen, have also been thought to be neuroprotective and involved in neurobiological processes involved in cognition, including memory [48]. Hormones are also proposed to intersect with other biological processes (e.g., inflammation, vascular risk) that may further impact cognitive functioning [49]. From a social perspective, recent research in European participants have proposed that there have been changes in the differences in cognition found between men and women based on gender differences. Specific cognitive abilities were found to vary systematically between men and women across birth cohorts and regions, which were associated with changes in living conditions and cognitive stimulation taking place over time [50]. The authors theorized that women benefited more from these societal improvements than men, as evidenced by increased gender differences in episodic memory, decreased gender differences in numeracy, and elimination of gender differences in category fluency. Further, the level and type educational experiences of men and women has historically been different, typically with women receiving less formalized education than men. Therefore, the differences seen within the current study could be related to these historical differences. Notably, sex and gender are inextricably linked, and it is likely an intersection of both biological and social factors that influence sex and gender differences in cognition. Taken together, the sex differences observed in the current study highlight the importance of developing sex/gender related norms.

### Finding 3: No sex difference in neuropsychological scores over time

Regarding sex differences in cognitive change over time, sex differences in rates of cognitive change were not observed. That is, males and females showed similar stability in neuropsychological scores across time. This finding is consistent with many longitudinal studies of healthy aging where there were no sex differences in rates of cognitive change over time [12,13,26,46]. Sex did not determine the rate of cognitive decline in a systematic review of older adults between the ages of 60–80 years of age [12], nor were there any differences in decline in one of the most robust samples to date from the UK Biobank, which was inclusive of participants starting from age 45 [22]. A minority of longitudinal studies have found sex differences in rates of cognitive decline, particularly in older adult samples. There have been mixed results for the rate of decline in males and females, which are dependant on the cognitive domain being measured. For example, global cognition, mental status, perceptual integration,speed, spatial ability, general information, and verbal memory [16,51]. However, these differences may result from the diversity in samples and measures used to assess these differences.

### Limitations

There are several limitations to the present study. In the current study, there were fewer female participants and greater male participant attrition. There are likely fewer female participants because the main project is about Parkinson’s disease, which has a higher prevalence in males. Nonetheless, this created unequal groups for comparison. Likewise, while there is often some level of attrition in longitudinal studies, the level of attrition in male participants was greater than female participants, which could lead to some inaccuracies or biases in the final results.

Additionally, the current study examined a group of participants that had a wide range of ages, which necessitates caution when interpreting stability of cognition over time and the stability of sex differences. Future studies could focus specifically on older adults as well as include additional interactions to examine time, age, and sex. This study was limited to the data collected by the PPMI. As data were only available for biological sex, the effects of gender and cognition were not specifically and separately examined in the current study. It will be important to examine both sex and gender in future research. Further, the sample of PPMI participants was largely white/Caucasian (92% of participants at baseline) and there were limited participants of other races/ethnicities, which were not well defined (i.e., “Black”, “Asian”, or “Other”). There is an urgent call to recruit more diverse samples in aging research as it has been found that underrepresented groups can be disproportionately excluded from participation in research studies, despite some groups having higher risk and burden of age-related diseases [52]. Participants included in the present study met criteria for PPMI’s healthy control cohort. As such, some of the participants scored below 1.5 standard deviations below the mean on a single neuropsychological measure at a single time point (e.g., MoCA score < 26). Although specific research consensus definitions have been created for groups along the trajectory towards age-related diseases, such as subjective cognitive decline [53], and mild cognitive impairment [54], there is less consensus on the definition of “normal” or “healthy” participants. It is important to note that the delineating factors between healthy and subjective cognitive decline are still underexamined in larger databases, therefore it is likely that there are participants within the current sample that may meet criteria for subjective cognitive decline. Further, there are multiple drivers of cognition and variation that may have influenced the current results. Factors like premorbid intelligence and cognitive reserve, which have influence over the trajectory of cognition, were not assessed in the current sample. Variance in these factors can influence the results by ‘skewing’ the data or unfairly biasing the samples. Similarly, there were baseline differences between the groups on the cognitive measures, and these differences may influence the observed changes and impact of the results and conclusions drawn. Further, neuroimaging data was not included in this analysis. Future research investigating sex/gender differences in brain changes in the healthy aging and sex/gender differences could be conducted utilizing data from the healthy control group data from PPMI (e.g., magnetic resonance imaing, diffusion tensor imaging, SPECT). Decisions about the inclusion/exclusion criteria for the healthy control participants were determined a priori by PPMI investigators. The implication is that the overall findings must be interpreted in this context, which resulted in a more restricted range of possible scores on neuropsychological measures. All available data for healthy control participants were inlcuded in the present study to maximize the study sample size.

## Conclusion

The present study revealed stability in cognitive functioning over five years. Although there were sex differences in neuropsychological scores on several measures consistent with the literature, the rates of change in these scores over time did not systematically differ between males and females. The current study contributes to a growing body of literature specifically examining sex similarities and differences in cognition and aging in healthy adults.

Future research should continue to examine the effects of sex and gender on healthy aging using the consensus definitions in the literature. As there was great intraindividual variability in healthy aging trajectories, it will also be important to continue to examine factors that may predict individual changes in cognition over time.
